# Effect of yoga in medical students to reduce the level of depression, anxiety, and stress: pilot study (Goodbye Stress with Yoga GSY)

**DOI:** 10.1186/s12906-024-04496-0

**Published:** 2024-05-24

**Authors:** Shalini Chauhan, Ann Mary Babu, Dahabo Adi Galgalo, Csaba Melczer, Viktória Prémusz, István Karsai

**Affiliations:** 1 Doctoral School of Health Sciences, Faculty of Health Sciences, University of Pécs, Vörösmarty u. 4, Pécs, Pécs, H-7621 Hungary; 2 Physical Education and Exercise Centre, Medical School, University of Pécs, Pécs, Hungary; 3 Institute of Physiotherapy and Sports Science, Faculty of Health Sciences, University of Pécs, Pécs, Hungary; 4 Institute of Psychology, Faculty of Humanities and Social Sciences, University of Pécs, Pécs, Hungary; 5 János Szentágothai Research Centre, Physical Activity Research Group, University of Pécs, Pécs, Hungary

**Keywords:** Yoga; mental health, Stress, Depression, Anxiety

## Abstract

**Introduction:**

Globally medical students reported high level of stress sensitivity, stress intensity and depression or anxiety. Yoga is proven to be a one of the most effective anxiolytic tools. The current study specifically designed to investigate the effect of yoga intervention on the level of stress, depression, and anxiety of medical student at the University of Pécs.

**Methods:**

Twenty-eight medical students from the University of Pécs, with an average age of 23.54 ± 4.36 years and a BMI of 22.42 ± 3.42 kg/m^2^, participated in a 10-week yoga intervention. In the current study, the DASS-21 was employed to gather information on stress, depression, and anxiety, while self-reported health and quality of life were assessed using the WHOQOL-BREF Questionnaire. The Shapiro-Wilk test was employed to examine the distribution of the data. The choice between the paired sampled T-test and the Wilcoxon signed test was determined based on the distribution of the data.

**Results:**

The intervention group exhibited a mean and standard deviation of depression pre- and post-yoga 10.14 ± 10.60 and 7.21 ± 8.56, similarly the values for anxiety were 8.57 ± 10.09 and 5.51 ± 7.42, and for stress values were 12.79 ± 10.73 and 9.64 ± 9.71 respectively. Notably, this outcome attribute to a significant in decreased depression (*p* = 0.019), anxiety (*p* = 0.049) among the participants.

**Conclusions:**

Introduction of Yoga Intervention significantly decreased in levels of depression and anxiety. By this current study we were able to confirm the necessity of Yoga Intervention with our primary survey.

**Supplementary Information:**

The online version contains supplementary material available at 10.1186/s12906-024-04496-0.

## Introduction

**Fig. 1:**
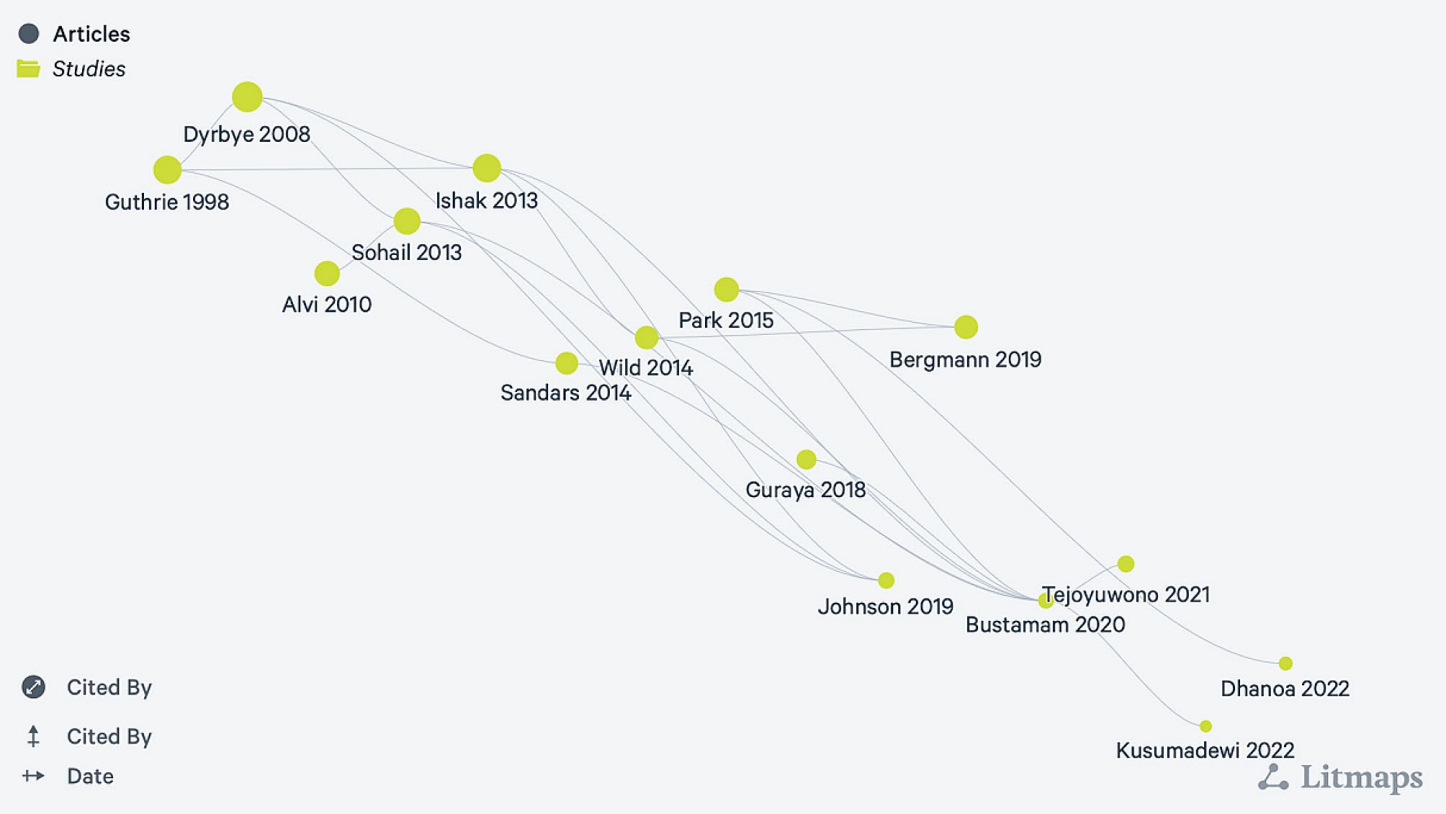
[7]. Studies illustrate the prevalence of stress, depression, and anxiety in medical students

University students faced quite a demanding transition into early adulthood from adolescence [1]. This brings major psychological and physiological changes, including elevated stress [2]. During this period, students feel independence by leaving home, lacking parental supervision, changing in close relationships, adjusting to culture with new people, and facing many situations that create stress [1]. There are a variety of contributing factors attributed to the stress experienced by college students [3]. Evidence shows that generally medical and dental students experience a higher level of stress symptoms, especially during their education. Based on the American Psychological Association, around 87% of university students reported education as their primary source of stress [4]. There are many studies that state that the level of stress in medical students is much higher (Fig. 1.) [5]. Despite Education, there are other factors also contributing to high levels of stress in university: intense academic competition, excessive demands on coping abilities emotional, physical financial, and social terms, and a highly competitive and continuously demanding environment [6].

It is proven by many studies that medical students have more signs of depression or anxiety, stress intensity, and stress sensitivity [8], However, the literature does not agree with the explanation of stress sensitivity, most approaches implicitly agree that different people react to stress in different ways. Those who are sensitive to stress require less exposure than those who are not [9]. These studies have valid explanations to state that the important challenge for the education institutions of these medical and dental studies is to deliver specific student emotional support along with different kinds of stress management techniques as well as appropriate steps for prevention curriculum reforms [8].

Globally, stress is very common among medical students [10]. From different regions around the globe, medical students have been found at risk of mental disorders, psychological stress, and decline in life satisfaction. Evidence states that it’s one of the main causes of the high dropout rate among medical students [11]. Relevant research conducted in Sweden revealed that the prevalence of depression symptoms in medical students is 12.9% and the percentage of suicidal attempts due to stress is 2.7% of total students [12]. Another study conducted in Saudi Arabia reported a very high prevalence of stress in 57% of total medical students [13]. Understanding the seriousness of this issue is crucial for comprehension and solution findings. It is important to state that this not only affects students’ academic achievement but also affects their health. By prioritizing the mental health and well-being of students, universities and medical colleges can create a supportive environment that fosters academic success and promotes a healthy and fulfilling life.

The evidence suggests that yoga is a beneficial technique for reducing stress and promoting overall health and well-being [14–16]. It is an ideal practice for one’s sustainable happiness, it can help to conceptualize individual potential, develop resilience, and elevate eudaimonia pleasure [17]. Yoga has gained popularity for its effectiveness in helping and maintaining a healthy lifestyle. Numerous studies have demonstrated its efficacy, and it is widely utilized as a form of mind-body therapy in the treatment of various clinical conditions, including cancer, eating disorders, hypertension, and pain relief etc [18–21]. To understand more facts about yoga it is important to understand the basic concept of Yoga.

Yoga is originated thousands of years ago; yoga is acknowledged as a form of mind-body medicine. The term “yoga” is derived from the Sanskrit root YUJ which signifying “to unite” and “to join”. It also refers to a method through which any person can achieve the connection between the body and the mind to attain self-realization [22]. The primary objective of yoga is to develop the union of mind and body through the combination of exercise (asana), respiratory (pranayama), and meditation to attain psychosomatic harmony [23]. This the holistic approach to healing which addresses both external and internal aspects of your body. If you compare it to usual physical activity, it offers a very different experience which is less tiring and more enjoyable [23].

The most recent systematic review conducted articles from January 2014 to November 2018 [24], reported the positive effects of yoga on stress reduction in the healthy adult population. However, caution is advised in interpreting the results of this review because of limited number of studies included, but most importantly remission was reported in this review. Additionally, the included studies show the diversity of yoga and have more methodological problems to prove the effect of yoga considering the higher amount of stress exposure by this population. In the current study, we endeavour to employ a methodologically rigorous approach, and our paramount focus while conducting this study was to reduce potential bias.

The overall aim of this pilot study is to examine the potential role of yoga in the medical student’s life to reduce stress, depression, and anxiety levels and improve the quality of life. Within this umbrella, we have many goals. First, to examine the necessity of intervention and whether 10 weeks of yoga intervention will be beneficial for student’s overall mental health. Second, to investigate the variation in stress, depression, and anxiety levels before and after the yoga intervention. And third to examine individual perception of quality of life in the recent days of before and after yoga intervention.

## Materials and methods

### Study design

An interventional study was carried out with consecutive sampling using a self-administered questionnaire in paper-pencil format.

### Sampling

Data collection was carried out of the medicine students of the University of Pecs, Hungary between February 15 to 13 of May 2022. Participants were involved into the study by taking their verbal and written consent, and both male and female participants from diverse international backgrounds and not living with their family were participated in the study. The inclusion criteria for the study required participants to be currently enrolled in the Medicine Faculty of the University of Pecs. However, certain exclusion criteria were established. Students with specific health conditions, including recent injuries, chronic pain, congenital skeletal problems or significant arthritis, which could potentially pose safety concerns during participation in a yoga intervention, were excluded from the study.

All the participants who enrolled for the current research met the inclusion criteria and participated in the study, none met the exclusion criteria, additionally there were no dropouts among the participants.

### Study participants

All participants who registered in the physical training course with the name “Indian Yoga” at the University of Pécs, Hungary during the month of February -May 2022 were invited to participate in this current research. The research specifically included those students who expressed interest actively in the current study (*N* = 28) with an average age of 23.54 ± 4.36 years and a BMI of 22.42 ± 3.42 kg/m2. Each participant received comprehensive instruction and a detailed explanation of the study.

### Intervention

The research spanned a period of 10 weeks, from February 2022 to May 2022, with 90 min of yoga sessions, occurring once a week. Each yoga session followed a structured format, consisting of a different activity as shown in (Table 1). The development of the yoga protocol, named “GSY Goodbye Stress with Yoga Protocol,” was a collaborative effort involving a certified yoga trainer, medical researcher, and experienced yogis from India.

**Table 1:** Description of intervention

Sequences	Duration
Full body warm-up and light stretching exercise	15 min
Performing various yoga postures - including standing, sitting, prone and supine yoga postures (Asanas)	50 min
Breathing exercises (Pranayama)	10 min
Meditation	15 min

Each part of the above-mentioned specific intervention is followed by 3 min of Corpse Pose (Shavasana). Study participants were instructed to not eat two hours before the yoga session. The yoga session took place in the gymnasium hall located at the University of Pecs. Before each session, the hall underwent comprehensive cleaning and preparation process. The researchers provided yoga mats to the participants, ensuring that each mat was used exclusively by the same individual throughout the sessions. Yoga mats were fully sanitized and cleaned before and after the yoga intervention. The session was conducted by a certified yoga instructor with 7 years of teaching experience.

### Assessment scales

The variables of the research framework were analysed by a self-reported questionnaire. These questionnaires were filled out at the Yoga session before starting the intervention and after the 10 weeks of intervention. The questionnaire was used in paper pencil form and deeply explained before giving it to the participants to have more accuracy in the response. Sociodemographic characteristics were obtained by using questions regarding, age, weight, education level, country, source of funding, relationship status, practised yoga before, other physical activity, and BMI, psychosocial characteristics were assessed by measuring the domains of depression, anxiety, stress, and quality of Life.

#### Assessment of physical activity

To assess participant’s physical activity levels, enable comparisons and analysis, using both subjective and objective assessment.

Global Physical Activity Questionnaires were used for Subjective Assessment and ActiGraph was used for Objective Assessment. Global Physical Activity Questionnaire GPAQ is used in validated English [25] to collect information on physical activity participation in the five domains (work-related vigorous, work-related moderate, travel, recreational vigorous, and recreational moderate) as well as sedentary behaviours comprising a total of 16 questions. According to the Based on the GPAQ responses regarding activity intensity, duration, and frequency, the total MVPA was also calculated in MET-min/week. The Overall MVPA level was further categorised as low, moderate, and high according to WHO guidelines [25]. ActiGraph GT3X-BT is ActiGraphy’s flagship activity monitor, globally used by researchers to capture and document continuous, high-resolution data on physical activity and sleep/wake patterns. ActiGraph devices have demonstrated precision in estimating accurate PA in free-living environments. These devices have been employed in many large-scale epidemiological studies across the world [26]. These tools were used to serve as a baseline and outcome measure to evaluate the impact of the intervention.

#### Assessment of stress

To investigate the variation in stress, depression, and anxiety levels before and after implementing a yoga intervention.

Depression Anxiety Stress Scale DASS 21 was applied in English [27]. This scale is utilized to assess the stress, anxiety, and depression experienced by students prior to and following the Yoga intervention. It includes 21 items, these items are in 3 sets of self-reported instrument created to assess the emotional state of depression, anxiety, and stress. The DASS 21 adopts a dimensional approach to evaluate psychological disorders, rather than relying on categorical classifications. Each set Includes seven questions with total scores that range from Normal to extremely severe [27].

#### Assessment of quality of life

*To Analyse the Individual perception of quality of life in recent days before and after Yoga Intervention.*

World Health Organization Quality of Like BREF WHOQOL BREF tool is used in English version [28]. This is a self-administered questionnaire that includes 26 questions on the individual perception of the quality of life and health over the previous two weeks. These questions are separated into four domains (1) Physical health, (2) Psychological,3. Social Relationships and Environment. The scoring of the question is based on a 1–5 Likert scale where 1 represents “disagree” and 5 represents “Completely agree” [28].

### Ethical approval

The study was reviewed and approved by the Regional Research Ethics Committee as the Institutional Review Board Record number 9117- PTE 2022 University of Pecs. Prior to the initiation of any study related procedures, participants provided written informed consent, indicated by their signature on the Informed Consent Form. The current study adheres to the principles outlined in the Declaration of Helsinki.

### Data analysis

Statistical analyses were performed using SPSS 26.0 software (SPSS Inc., Chicago, ILUSA).

For the baseline data, descriptive statistics were used. Data were presented as percentage (%) and frequency (N) for categorical variables, while continuous variables were presented as mean ±SD. Shapiro-Wilk test was used to test the normality of the data. The Wilcoxon Signed Ranks Test was utilized to assess the mean differences between the pre- and post-data. Paired sample correlation was used to investigate the correlation in the pre- and post- data, and Spearman’s Rank Correlation test was also applied. The significance level of p˂0.05 was considered in each case.

## Results

### General characteristics

The major socio-demographic data of the study participants are shown in Table 2. The mean age of participants is 23.54± 4.36 years and a BMI of 22.42±3.42 kg/m^2^. The medium height of participants is 165 cm (155–188) and the medium weight of participants is 60 kg (48–98). 78.6% of female participated and 21.4% of the male were participated in the current study. 82.1% of the participants were living in the city at the time of the study. 42.% of the participants have scholarships for their financial support.

**Table 2:** Sociodemographic characteristics of the study populations

Characteristics	Frequency (%)
**Age Group**	
< 20	6 (21.4)
21–25	15 (53.6)
> 26	7 (25.0)
**Mean age (years)**	23.54 ± 4.36
**Gender**	
Female	22 (78.6)
Male	6 (21.4)
**Residence**	
City	23 (82.1)
County	3 (10.7)
Village	2 (7.1)
**Financial Support**	
Self-Funding	16 (57.1)
Scholarship	12 (42.9)
**Source of Income**	
Family	15 (53.6)
Scholarship	13 (46.4)
**Anthropometrics**	
Height (cm)	165 (155–188)
Weight (kg)	60 (48–98)
BMI (kg/m^2^)	22.42 (3.42)

*BMI- body mass index, cm- centimetre, kg-kilogram, kg/m2-kilogram/square meter

### Physical activity

Data was collected on the baseline activity level by using (Global Physical Activity Questionnaire, Table 3) and accelerometers (ActiGraph GTXbt, Appendix A) to compare the difference between subjective assessment and objective assessment. Participants reported higher levels of physical activity MVPA (Moderate-to-Vigorous Physical Activity) 483.39±470.91 min/week when assessed using GPAQ (Global Physical Activity Questionnaire) tool, compared to 275.25 ±128.06 min/week when measured using the ActiGraph GTXbt.

**Table 3:** Mean and standard deviation of (GPAQ) global physical activity questionnaire after 10 weeks of yoga intervention in participants

		Work			Transport	Recreation			MVPA total	Sedentary	
	*N* = 28	Vigorous	Moderate	MVPA		Vigorous	Moderate	MVPA			
Pre	Mean	0.36	109.28	110.00	216.90	61.40	33.50	156.40	483.30		473.80
	Median	0.00	0.00	0.00	200.00	25.00	5.00	95.00	405.00		480.00
	SD	1.90	475.50	475.50	193.60	99.60	41.10	200.30	470.90		174.40
	IQR -L	0.00	0.00	0.00	0.00	0.00	0.00	0.00	0.00		120.00
	IQR-U	10.00	252.00	252.00	840.00	360.00	120.00	720.00	2520.00		840.00
Post	Mean	61.42	170.70	293.50	304.28	112.67	82.50	305.85	601.40		440.30
	Median	0.00	0.00	0.00	180.00	40.00	15.00	15.00	205.00		480.00
	SD	180.70	677.80	767.65	363.63	182.57	147.03	430.84	987.35		190.00
	IQR -L	0.00	0.00	0.00	0.00	0.00	0.00	0.00	0.00		20.00
	IQR-U	600.00	3570.00	3570.00	1500.00	750.00	600.00	1530.00	3570.00		840.00
Z		-1.60	-0.84	-1.36	-0.85	-1.00	-0.87	-1.19	-0.64		-0.16
p		0.11	0.40	0.17	0.35	0.32	0.38	0.23	0.53		0.43

*N: number of participants, IQR -L: Interquartile range -Lower, IQR -U: Interquartile range -Upper MVPA: moderate to vigorous physical activity

Response of participants before and after yoga intervention on moderate vigorous physical activity (MVPA) in work domain changed from 110.0±475.5 min/week before yoga and 293.5±767.65 min/week after yoga respectively. Participants responded on MVPA in recreational domain 156.4±200.3 min/week and 305.85±430.84 min/week. Participants responded that they spend before and after the yoga intervention on average with active transportation, for example walking or cycling 216.9±193.6 min/week and 304.28±363.63 min/week. They spent 473.8±174.4 min/week and 440.3±190.0 min/week in sedentary activity. However, there is no statistical improvement reported after the yoga intervention in MVPA in work domain (*p* = 0.173), MVPA in recreational domain (*p* = 0.231), total MVPA (*p* = 0.525), transportation activity domain (*p* = 0.355) and in sedentary activity domain (*p* = 0.434).

### Changes in the perception of quality of life

Based on the World Health Organization Quality of Like BREF (WHOQOL-BREF, Table 4) which examined general quality of life (QoL), The mean and standard deviation of the physical health domain in the intervention group pre and post was 50.97±11.10 and 59.07±9.27, their response in the quality of life domain was 80.71± 17.62 and 82.14 ± 13.71, participants respond in Psychological health domain score was 56.79±9.90 and 68.25±6.18, their response in environment health domain score was76.16± 13.66 and 77.12± 13.92 and the response in social relation domain score was 62.48 ±23.22 and 71.07± 20.09 respectively.

**Table 4:** Mean and standard deviation of DASS 21 depression anxiety and stress 21 score and WHOQOL BREF of before and after 10 weeks of yoga intervention in participants

WHOQOL-Bref	Mean ± SD			
	Pre	Post	Change	*p* value
Physical Health domain score	50.97 ± 11.10	59.07 ± 9.27	-8.50 ± 12.56	0.001*
Quality of Life domain score	80.71 ± 17.62	82.14 ± 13.71	-1.43 ± 17.99	0.678
Psychological Health domain score	56.79 ± 9.90	68.25 ± 6.18	-11.46 ± 10.06	< 0.001*
Environment Health domain score	76.16 ± 13.66	77.12 ± 3.92	0.96 ± 15.12	0.060
Social Relation domain score	62.48 ± 23.22	71.07 ± 20.09	-8.59 ± 14.98	0.754
**DASS 21**	Mean ± SD			
	Pre	Post	Change	*p* value
Depression	10.00 ± 10.60	7.21 ± 8.56	2.83 ± 6.19	0.019*
Anxiety	8.57 ± 10.09	5.50 ± 7.42	3.07 ± 7.90	0.049*
Stress	12.79 ± 10.73	9.64 ± 9.71	3.14 ± 9.08	0.078

*Statistically significant difference between the yoga and control groups (defined as *p* < 0.05)

Students who participated in the yoga intervention showed statistical significantly improved in physical health domain (*p* = 0.001) and psychological health domain (*p* < 0.001). They showed improved in quality-of-life domain (*p* = 0.678), social relation domain (*p* = 0.754) and environmental domain (*p* = 0.060), but not statistically significant.

### Changes in stress, anxiety, and depression

Based on the (Depression Anxiety Stress Scale DASS 21, Table 3).

The mean and standard deviation of depression reported in participants before and after 10 weeks of yoga intervention for pre and post was 10.00 ± 10.60 and 7.21 ± 8.56, their anxiety reported in participants was 8.57 ± 10.09 and 5.50 ± 7.42, and participants stress reported before and after the intervention was 12.79 ± 10.73 and 9.64 ± 9.71 respectively. Depression and anxiety score significantly decrease after the yoga Intervention (*p* = 0.019 and *p* = 0.049). Stress score decreased after yoga intervention but not significantly (*p* = 0.078). Change reported in all measure stress anxiety and depression after 10 weeks of Yoga Intervention. These findings collectively indicate that 10 week of yoga intervention have positive effects on medical students, leading to improvements in their depression, anxiety, and stress levels.

## Discussion

The current study findings demonstrated that yoga is a potential technique to reduce depression, anxiety, and stress and to improve the quality of life in medical students. According to Evidence depression is the primary cause of disability on global scale, and stress, anxiety depression is the significant contributor to the worldwide burden of disease [29]. Many people experience comorbidities that certainly affect their quality of life [30]. Education in medical school is considered to be distinctive the most stressful, which certainly causes an increase in stress, anxiety, and depression [29]. Medical universities usually struggle to introduce student support programs that can help them reduce stress, depression, and anxiety and improve their quality of life [31].

The present study evaluates a 10-week yoga intervention among medical students. The BMI, anxiety, stress, depression, and perception of quality of life were evaluated. A significant decrease in depression and anxiety was observed following 10 weeks of yoga intervention compared to baseline levels. Students responded and showed significant satisfaction in all four domains: psychological health, environmental health, physical health, and quality of life. This preliminary investigation suggests a positive effect of yoga on the student’s stress support management.

### Physical activity

In our current pilot study, we reported that during the 10-week of yoga intervention medical students did not significantly (*p* = 0.525) change their physical activity habits as participants don’t change their regular lifestyle according to GPAQ data except the yoga sessions. According to this finding the improvement in mental status (depression and anxiety) and quality of life may be resulted by yoga and not by other lifestyle change.

Including yoga and other form of physical activity in their assessment, have moreover inconclusive results for physical activity outcome assessment [32]. This observation is consistent with an experimental study conducted in India by U.S Ray at al., 54 participants of this study was a group of engineering fellowship course trainees. The primary aim of the study was to observe the effect of yogic practices on physical and mental health of the participants.

This study indicated that the yoga group maintained their physical activities in a mixed group way which means when yoga and other type of physical activity performed by the participants [33]. This kind of results are not clear to explain the effect, because the presence of yoga and other influencing factor parallelly. In our current study we explained that the improvement in mental status (depression and anxiety) is due to yoga intervention.

Our study also highlighted that there should be more objective assessment of PA to have more reliability of results reported. In our study participants significantly overestimated their activity level, when comparing objective and subjective data. We did not find any previous study that examined the effect of yoga and the level of the physical activity of the medical students or and other sample, and which can analyse the comparison between the objective assessment and subjective assessment of PA to have more reliability of results reported.

### Stress

Our study found that the 10 weeks of yoga intervention changed stress (3.14 ±9.08) *p* = 0.078 but data were not statistically significant, this finding is consistent with the study done by Allison R. Bond, that 11 weeks of yoga intervention, with three times a week shows changes in perceived stress (-0.05±0.62) but not statistically significant (*p* = 0.70) [34]. This is a contrast to previous randomised control trial study done on individualised yoga which shows that yoga intervention is statistically significant (*p* < 0.01) in reducing stress score. But the participants of this trial were general population with different age group [30]. This finding suggests the effectiveness of intervention to reduce stress score. Other studies have demonstrated that yoga decreases the level of stress in college students which leads to positive effects on their psychophysiological level [35]. In the current study post data were collected during the exam period, and in which typically students has the higher level of stress [36], we concluded that the yoga intervention helps them to manage their stress level during exam period which also shows the necessity of intervention during exam period to improve individual’s productivity.

### Depression and anxiety

Studies on yoga demonstrated the effect of yoga in-home practice, it states that in terms of the broader community, it might have several benefits associated with mental health. With additional health benefits reduction of depression and anxiety is more over-reported from these studies [30]. Studies that explored the effects of yoga and meditation on the depression and anxiety differed in type of population, study design or the length of interventions. However, there were many similarities with the current study. In randomised controlled trial of mindfulness versus yoga, study showed that in the yoga group compared with control group depression and anxiety scores decreased significantly (*p* < 0.01) [37]. Study by Burns et al. was conducted on the effect of meditation for two semester period consistently on college students. This study focused on anxiety and depression, participants showed significant decreased in the depression (*p* < 0.000) and anxiety score (*p* < 0.0o7) [38]. In the above studies we can see that despite have the different study design and different period of intervention all the studies showed that yoga can significantly decrease the depression and anxiety score. The present study also showed the statistically significant to reduce the depression (*p* = 0.019) and anxiety score (*p* = 0.049) based on Depression Anxiety Stress Scale DASS 21.

### Quality of life

According to the World Health Organization (WHO), quality of life can be defined as an individual’s perception about his/her life in the context of value system, surroundings and culture in which they live and in connection or relationship to their goals, standards, concerns and expectation [39]. The decrease in quality of life possible contribute to sleep disturbance fatigue and medication abuse and to elevate stress [40].

Our study demonstrated that 10 weeks of yoga intervention can improve perception quality of life, for example, participants reported significant improvement in physical health domain score (*p* = 0.001), psychological health domain score (*p* < 0.01) and environmental health domain score (*p* = 0.06), these findings is consistency with study done by Oken and colleagues (2006) which evaluated the effect of yoga on various populations where they found an improvement in the perception of quality of life, for example, physical ability (balance, flexibility) (*p* = 0.01), social functioning (*p* = 0.06), and physical functioning (*p* = 0.015) in the 90 min /per week yoga intervention for 6 months. However this result is reported in the older adult age group (65–85 years) [41]. In summary we concluded that despite any age group yoga intervention has the potential to improve the quality of life in all the age groups.

### Limitations

The current study has several limitations. There was a lack of control group, including control group would help to ascertain whether the outcome of research or observed changes were due to intervention of other factors. A small sample size reduced the statistical strength of the research. In addition, we lack of follow up data on the psychological well-being students as current study was only for one semester duration. Another possible limitation which should take into consideration is that students voluntarily chose to enrol in study courses and were therefore self-selected group. Thus, students who participated in the research may have possessed unique interests and characteristics that differed from those of other students.

This suggests that further studies are required with an active control group, a larger sample size, and with long duration of intervention weekly to have more clarity and generalize the findings.

## Conclusion

Beyond the substantial interest in the intervention, our pilot study was able to provide validation for the efficacy and necessity of the yoga intervention with our preliminary survey. It demonstrated that 10 weeks of yoga intervention can result in a significantly reduction in the perceived level of anxiety, depression, and stress.

Despite collected the post research data during exam period participants shows reduction in stress and along with this it shows yoga can significantly improve the quality of life.

At the same time, further detailed analyses with large sample size and a more objective research design are required to strengthen the findings.

### Electronic supplementary material

Below is the link to the electronic supplementary material.

Supplementary Material 1

## Data Availability

The datasets used and/or analysed during the current study available from the corresponding author on reasonable request.
