# Supplementary material for: Effect of yoga in medical students to reduce the level of depression, anxiety, and stress: pilot study (Goodbye Stress with Yoga GSY)
Source: BMC Complement Med Ther. 2024 May 24;24:203. doi: 10.1186/s12906-024-04496-0 (PMC11127401; doi:10.1186/s12906-024-04496-0)
Supplement: Supplementary file 1 — Supplementary Material 1 [file 12906_2024_4496_MOESM1_ESM.docx]

|  | **Light min/week** | **Moderate** | **Vigorous** | **Very Vigorous** | **Sedentary** | **Total MVPA** | **Steps/Day** |
| --- | --- | --- | --- | --- | --- | --- | --- |
| **Mean** | 988.50 | 244.67 | 28.33 | 2.25 | 8936.67 | 275.25 | 5191.30 |
| **Median** | 971.00 | 236.50 | 15.00 | .00 | 8938.50 | 255.00 | 4869.81 |
| **SD** | 369.77 | 111.98 | 50.61 | 5.59 | 478.05 | 128.06 | 2180.58 |
| **IQR-L** | 817.25 | 156.75 | .25 | .00 | 8722.50 | 160.25 | 3316.63 |
| **IQR-U** | 1227.00 | 355.00 | 29.25 | .00 | 9118.00 | 392.75 | 6925.13 |

**Appendix A. Mean and Standard deviation of ActiGraph GT3X-BT before 10 weeks of Yoga intervention in Participant**

*IQR -L: Interquartile range -Lower, IQR -U: Interquartile range -Upper, MVPA: moderate to vigorous physical activity.
